# The role of stigma in the acceptance and disclosure of HIV among recently diagnosed men who have sex with men in Australia: A qualitative study

**DOI:** 10.1371/journal.pone.0224616

**Published:** 2019-11-08

**Authors:** Jade E. Bilardi, Alana Hulme-Chambers, Marcus Y. Chen, Christopher K. Fairley, Sarah E. Huffam, Jane E. Tomnay

**Affiliations:** 1 Central Clinical School, Monash University, Victoria, Australia; 2 Melbourne Sexual Health Centre, Alfred Health, Victoria, Australia; 3 Department of General Practice, University of Melbourne, Victoria, Australia; 4 Centre for Excellence in Rural Sexual Health, University of Melbourne, Victoria, Australia; New York Blood Center, UNITED STATES

## Abstract

**Background:**

Our primary study aimed to explore the experiences of men who have sex with men (MSM) recently diagnosed with HIV and their partner notification practices. Themes relating to acceptance, and disclosure of, their HIV status strongly emerged during analysis in our larger study and are reported separately here.

**Method:**

Fifteen MSM participated in semi-structured interviews by phone or face to face about their experience of a recent HIV diagnosis. In this paper we report on how they received and accepted the diagnosis, who they disclosed their diagnosis to and what is needed to improve support for MSM recently diagnosed with HIV.

**Results:**

MSM’s reactions to their HIV diagnosis ranged from shock, devastation and anger to a calm acceptance and feeling HIV would not have a significant impact on their lives. MSM who reported strong social support networks, or knew others with HIV, seemed better able to cope with and accept their diagnosis than those with fewer support networks. Due to prevailing stigma around HIV, most MSM were very selective about who they disclosed their status to, often only telling partners perceived to be at risk but no, or only few, close friends. Regardless of how well men accepted their diagnosis, most did not disclose their status to family members for fear of rejection or causing distress due to ideologies based on outdated information about HIV.

**Conclusion:**

The prevailing stigma around HIV can have a significant impact on MSM’s acceptance of, and willingness to disclose their HIV serostatus to others, and consequently the levels of professional and social support they receive. HIV-related stigma needs to be addressed through community campaigns which better educate the wider population about the current state of HIV prognosis and treatment.

## Introduction

In Australia, the largest proportion of new HIV diagnoses are attributable to men who have sex with men (MSM).[1] Acceptance and subsequent disclosure of HIV serostatus is a key component of the wider prevent, test, and treat context.[2] However, a range of individual, community, cultural and societal factors can make acceptance of HIV diagnosis and disclosure to family and friends, difficult and frightening.[3, 4] While the medical landscape of HIV prevention, testing and treatment has undergone rapid advances globally over the past decade[5], and the availability of anti-retroviral therapy means that HIV is no longer considered a ‘death sentence’[6], there remains reluctance to disclose HIV serostatus in the context of a new diagnosis and the partner notification (PN) process because of perceived risks and negative outcomes experienced when doing so.

The original aim of the original study was to explore the PN experiences of MSM following a recent HIV diagnosis; other results from this study have been reported elsewhere [7]. In exploring MSMs experience of receiving a positive HIV diagnosis and the partner notification process; it became evident the prevailing stigma around HIV can have a significant impact on their acceptance of, and willingness to disclose their HIV serostatus to others, specifically family and friends, and consequently impact on the levels of professional and social support they receive at the time.

HIV stigma remains a key confounding factor in efforts to prevent HIV transmission.[5, 6]. The presence of HIV stigma has been shown to result in fewer disclosures, including to sex partners, healthcare workers and family members[8–11] as well as being an obstacle to accessing care and treatment.[12] Implications of non-disclosure include experiencing psychological distress,[13] engaging in behaviours with potentially harmful outcomes (such as substance misuse), and inconsistent compliance with treatment recommendations.[5] In a recent Australian study by Bell et al., (2018) about unwanted disclosure of HIV, participants described their struggles to retain agency over their identity in response to negative reactions to their HIV serostatus.[4] The study highlighted how attempts to retain or regain control of one’s identity post HIV diagnosis is largely an individual exercise that has little impact on the context in which people living with HIV negotiate work, social and personal lives.

In Australia, there is limited research exploring the experiences and factors influencing MSMs acceptance and disclosure of their HIV sero-status. With MSM continuing to be at higher risk to HIV exposure in Australia than other population groups,[14] it is important to understand what may constrain MSM in accepting and disclosing their HIV serostatus to others, including partners, family and friends. The findings relating to these themes from our primary study are reported here separately.

## Methods

This study has been reported in accordance with the Consolidated criteria for reporting qualitative research (COREQ) guidelines.[15]

### Ethics

Ethical approval for this study was granted by the Alfred Hospital Ethics Committee, Victoria, Australia; Application Number 45–14 on the 10th June 2014 and The University of Melbourne, Victoria, Australia; Application Number 1442671.

### Theoretical framework

A social constructionist viewpoint informed the framework for this study. From a social constructionist viewpoint, there is no one true ‘reality’, rather people’s perceptions of reality–their thoughts, knowledge, language, perceptions and beliefs—are socially constructed and shaped by the cultural, historical, political and social norms operating at the time and within that context.[16] From this perspective, each person’s experience or ‘reality’ of being diagnosed with HIV and subsequently undertaking partner notification will differ and what is important is not the accuracy of their accounts but rather their lived experiences and personal realities. People’s ‘reality’ can be greatly influenced by the cultural and social meanings attributed to an illness or condition, particularly if the condition is stigmatized.[17] Sexually transmitted infections (STI), and in particular, HIV, have considerable negative stigma associated with them[8, 18, 19] which can in turn impact on the ways in which people cope with, understand, manage and disclose their HIV diagnosis.

### Method, research team and reflexivity

Semi–structured interviews were undertaken with MSM as they allowed men to tell us their lived experience of being diagnosed with HIV while also allowing us to explore key areas of clinical interest i.e. partner notification practices. All interviews were undertaken by JT, an experienced sexual health researcher with over 15 years’ experience as a partner notification officer for the Department of Health and Human Services, Victoria, Australia. JT had no prior relationship with any of the participants and they were informed the study was being undertaken in an effort to better understand the experiences of MSM recently diagnosed with HIV and their partner notification practices. JB is a senior research fellow with a doctorate (PhD) in public health and specialises in social health research in the area of sexual and reproductive health. AHC is a research fellow and experienced social health researcher with a doctorate in (PhD) health promotion, specialising in qualitative approaches to sexual health research.

### Recruitment

Men were purposively invited to participate in the study. To be eligible for the study men had to be over 18 years of age, identify as MSM, reside in Australia and have been diagnosed with HIV in the past 12 months. Men were recruited from three health services across Melbourne, Victoria, Australia: the Melbourne Sexual Health Centre (MSHC), Victoria’s largest sexual health centre; Alfred Health, a major hospital with a HIV outpatients clinic; and Prahran Market Clinic, a metropolitan private general practice with a focus on sexual health and specifically the lesbian, gay, bisexual, transsexual/transgender, and intersex community. Participants were also recruited through the Phoenix Group, a workshop run by Living Positive Victoria (livingpositivevictoria.org.au) which aims to provide support to people recently diagnosed with HIV, and through partner notification officers (PNO) at the Victorian Department of Health and Human Services. Posters and flyers advertising the study were placed in the waiting rooms at each health service. Nurses, doctors or PNO’s provided information to eligible patients and asked if JT could contact them to discuss the study further. Consenting patients provided a contact number and JT contacted them directly to inform them further about the study and arrange a mutually convenient interview time and place.

### Data collection

Participants were interviewed either face to face or by phone. All interviews were conducted between October 2014 and June 2016.

#### Consent, confidentiality and the interview process

Participants interviewed face to face were provided with the plain language statement (PLS) and consent form prior to the interview commencing. Participants interviewed by telephone either received a copy of the PLS from the recruiting clinic or were emailed or sent the PLS prior to the interview or were read the PLS by JT at the time of the interview. Verbal consent was provided over the telephone and the consent form signed by JT on the participant’s behalf. The interview schedule was informed by current literature and the clinical expertise of the research group and included 21 mainly open ended questions on MSMs: Views on HIV, risk taking behaviour and meeting partners, HIV testing history, advice at the time of diagnosis, partner notification practices and experiences and their views on possible partner notification resources. Due to the sensitive topic area, difficulty recruiting participants and MSM’s concerns about confidentiality, no demographic data were collected other than participant’s first names and contact numbers. Data pertaining to MSM’s methods of meeting partners, partner notification practices and experiences and their views on a HIV PN website have been published previously.[7] All interviews were digitally recorded and transcribed verbatim. Participants were reimbursed AUD $40 for their time and any associated travel costs in attending an interview. Some men chose not to accept the payment due to confidentiality concerns (i.e. they did not want to provide contact details).

### Data pre-analysis & analysis

All interviews were read and analysed independently by JT, JB and AHC. Throughout the data collection period JT, JB and AHC met regularly to review and discuss themes emerging from the interview data. After 7 interviews were complete, an interim thematic analysis[20] was undertaken whereby all three researchers independently read and manually coded the transcribed interviews, using primarily a segmented approach,[21] and grouped the codes into broader themes to develop a coding framework. At this point the researchers met to discuss and compare the emerging themes and interpretations (cross coding technique).[22] Themes were derived both deductively from current literature, clinical practice and the interview schedule questions and inductively from recurring themes in the data itself.[23] At this meeting, the researchers identified two emerging themes around MSM’s views on HIV before and after diagnosis and their partner notification practices and experiences that required further exploration, and nine additional questions or probes were added to the interview schedule for future interviews. At the completion of 15 interviews, the same process of cross coding was employed with researchers meeting face to face to discuss in depth their analysis and refined coding and thematic frameworks. Despite some variation in coding frameworks, principally relating to coding language or wording, there was strong consensus among the three researchers around the major themes/sub themes and interpretation of data, with no notable differences evident. At this time, it was agreed that no new themes were arising from the data and data saturation had been met. JB collapsed the three researchers’ individual coding frameworks into one final agreed version, before importing the data into N-Vivo 11 for data management and undertaking a final read of all manuscripts and review of the coding and thematic analysis.

## Results

Of the 39 eligible men interested in participating in the study, a total of 15 were interviewed. The remaining 24 men were either not contactable after repeated attempts (n = 21) or did not attend for the agreed interview or respond to follow up messages (n = 3). No further men were invited into the study after saturation point was reached at 15 interviews. Of the 15 participants, three were referred from MSHC, one from Alfred Health, two from Prahran Market Clinic, one from the Phoenix Group and eight from PNO’s. Interviews took one hour on average. Of the 15 men, 10 reported being tested for HIV regularly in the past, and had received a negative test result within the past 12 months. Of the remaining five participants, four had sought testing either due to high risk sexual behaviour or symptoms of HIV. One participant had sought testing for immigration purposes.

Four key themes arose from the data: 1) Finding out they were HIV positive; 2) Accepting their diagnosis; 3) Disclosing their HIV serostatus to others; and 4) What HIV positive MSM need.

### Finding out they were HIV positive

The majority of men in this study were diagnosed with HIV at specialist services. Most men reported engaging in condomless sex with casual and/or regular partners prior to their diagnosis, and many men reported high risk sexual behaviors with casual partners they largely met online or through dating apps for once off sexual encounters.

Men’s reactions to their diagnosis varied from feelings of shock, distress or anger to calm acceptance and a feeling it was ‘no big deal’.

I'm a healthy person, I eat well and I look after myself and my fitness. So the only boundary for me would be getting around it in my head and to be quite honest, I've accepted it. Actually, people have been very surprised by my reaction. It's like well okay, this has happened. It's just another challenge in life. (Participant 10)

While some men accepted their diagnosis quite well, most men reported feeling shocked, numb or distressed at the time of finding out they were HIV positive.

…I was shocked that time, whatever he asked I just couldn't think …when I had the result I was just like kind I felt I got pain. I cannot cry, I cannot smile, I'm just blank. (Participant 4)

…I knew then straight away. He [doctor] didn't have to say it. I just went numb…nothing—not everything was sinking in … (Participant 8)

The few MSM who felt they had always engaged in safe sexual behaviors felt particularly perturbed and perplexed by their diagnosis.

…it was twice in my life that I've probably had unprotected sex and out of one of those two times I was just lucky enough to get it… .So obviously this time it was, yeah, a pretty big shock for me because I wasn't really expecting, yeah, HIV. (Participant 2)

For a number of men, their concerns around their HIV diagnosis seemed to center less on their actual diagnosis and more on the impact it could have on their visa or immigration status or their relationship with their partner.

…I think both, we were more concerned about the consequences with Immigration, because it's—it can be a ground to refuse your Visa, being HIV positive, rather than the disease itself, because he also—he's negative. (Participant 14)

Overall, most men, regardless of how they felt about their diagnosis, did not want to attribute blame for the transmission of HIV, feeling sexual partners would not have knowingly have transmitted HIV.

I don't want to attribute blame or anything, because I don't think—maybe I'm just naïve or whatever. But I don't like to think anyone would have done that on purpose. (Participant 9)

A couple of men however, who felt their partners had not been honest with them about their HIV serostatus or risk of transmitting HIV, reported feeling hurt and angry.

…so I kind of accused him, like why you do this on purpose? …You can feel that anger because you, if he knew he was passed to you, he could tell me and I can get PEP in 72 hour and I won’t get infected. (Participant 1)

### Accepting their diagnosis

Men reported varying reactions in the days, weeks and months following their diagnosis. Of the handful of men who seemed to accept their diagnosis quite well, a few reported little bearing on their day to day lives, particularly where they did not feel HIV would impact significantly on their lives.

…when I was diagnosed, I honestly wasn't too fazed about it because I knew what treatment methods and everything were out there. I didn't see it as some huge, massive, life-changing deal like a lot of people do. (Participant 15)

Others however—including a couple of men who seemingly accepted their diagnosis quite well initially—reported going on sexual ‘rampages’, drug and alcohol binges, breaking up with their partners or making significant changes to their sexual or lifestyle behaviours as a result of their diagnosis.

…a lot of people I know, myself included, when you find out that you've got HIV you kind of go on a bit of a rampage and you kind of go, fuck it, it doesn't matter anymore. I went on a bit of a bender when I found out. My boyfriend [unclear] he got deported back to America, he couldn't get sponsored so he moved back to America. After that I just went, fuck it, I had big time benders every weekend, just fucking a lot of people unprotected. So yeah, I feel like that's where a lot of the damage can come from. (Participant 3)

A couple of men also reported feeling depressed in the weeks and months following their diagnosis, concerned about the impact HIV would have on their lives and particularly their future relationships.

…this is not only my story, this is a story of many HIV gay people…they go into depression, they commit suicide…. So this is something that should be taken seriously I would say. (Participant 13)

For a number of men, the reality of having HIV very much differed from the idea of having HIV.

…[there’s] kind of this, an illusion about what it—so when guys are out there having unprotected sex and they're thinking oh I could get HIV. But it's fine, I can take a pill and it won't be an issue …But when the reality of that hits for somebody, it's huge and it's so different to what their kind of fantasy or their belief about what it means to be HIV positive is. . . . (Participant 7)

Overall, there were a number of factors that seemed to influence how well men accepted their HIV diagnosis. MSM who reported strong social support networks, good levels of knowledge around HIV (hereafter referred to as HIV health literacy) and/or knew others with HIV seemed better able to cope with and accept their diagnosis than MSM who knew little about or had out of date ideas about HIV and/or did not have strong support networks.

I was quite content with it. Previously, I've had friends who [have been] living with HIV for a few years now and on regular treatments, and I've seen and heard their stories. So I knew that I was going to be perfectly fine. I wasn't concerned with myself at all. (Participant 15)

I feel like I haven't had that traumatic an experience from it because I knew the support is there, I had some knowledge of it already—of how people can live with it and what treatments are available. (Participant 11)

These men often felt HIV was a manageable illness, not a ‘death sentence’ these days and differed little from any other medical condition requiring regular treatment.

HIV these days isn't a death sentence. It's not hepatitis C. It's not diabetes. It's not something that people die every day from. It's just a condition. (Participant 6)

### Disclosing their HIV serostatus to others

#### Stigma and lack of HIV knowledge

Almost all men spoke of the prevailing stigma surrounding HIV, which for many, impacted on their disclosure and acceptance of being HIV positive. MSM often attributed the prevailing stigma to the ‘grim reaper’ campaigns of the 1980’s and continuing misconceptions around HIV treatment and transmission and a lack of up to date information in both the broader and medical community around the advances in HIV treatment and prognosis.

…the problem is there has been no further education on that for people…I'm talking from close friends of mine who also have it [HIV], who have been [brandished] from a stigma that was drawn from knowledge or advertising, education from 30 years ago. If you look back to the '80s and things like that (Participant 10)

I think people don't know what's happening in 2016 with HIV. I think there is still a lot of those ads from the '80s, '90s, and the people got really shocked. So I think a lot of people think that way like it's still really—like, really bad, to the point of stepping back when you meet somebody with HIV. (Participant 14)

#### Disclosing serostatus to others

Men’s fear of rejection or judgment due to the prevailing stigma meant most were very selective about who they told about their diagnosis. Aside from notifying partners they felt were at risk, most men had either not told anyone, or only told a few friends about their HIV diagnosis.

Participant 8: That's the big thing that I was worried about—how my family would react and my friends.

Interviewer: That you might get rejected?

Participant 8: Yeah, the rejection.

Only a couple of men reported telling everyone they were HIV positive, generally via social media, as a means of freeing themselves from the judgement and stigma.

It's like the more you hide from it, the more you hide from them, the stigma will go on. If you stand proud and say you know what, I've got it and you education [sic] people, it's okay now. There is medication that will make us almost non-contagious. Everyone thinks of the Grim Reaper. Everyone thinks HIV is AIDS. But AIDS isn't HIV, it's different. (Participant 6)

While most MSM reported high levels of support from those they had told, a few men reported instances of discrimination, rejection or abuse as a result of disclosing their HIV serostatus.

…there were one or two that were really disgusted—like how can you do this to me? But these two people I never slept with …They got to the stage where—one of them got to the stage where he was actually abusing me and stalking me. (Participant 8)

A couple of men also reported poor experiences with medical professionals.

I had a really, really, bad experience at [a health service], because the nurse, she told me that it was the first time she had to deliver bad news and that she had a minimum training, but she was inexperienced. She made a comment saying that–‘Don't worry, HIV is quite—unfortunately quite common amongst gay people’…Then she took me to the waiting room where there were other patients…She told me, wait here. She said to other nurses, in front of other patients, and I could hear that, that ‘Oh, his results came back positive for HIV’. (Participant 14)

Many men, regardless of how well they accepted their diagnosis or their levels of HIV health literacy or support, did not feel able to disclose their serostatus to their family for fear of rejection or causing them distress as a result of their outdated perceptions of HIV or negative attitudes toward those living with HIV.

I still haven't told my family. They're very much old-school medical people and I don't think they would handle it too well because they still think HIV as a death, basically. It'll take some time for me to educate them before I tell them, I think, my family. (Participant 15)

### What HIV positive MSM need

Men suggested a number of ways to improve support and minimize adverse outcomes for people newly diagnosed with HIV, including providing up to date HIV information and resources at the time of diagnosis, counselling and support opportunities both at the time of diagnosis and in the months following, and safe social avenues and services to meet other HIV positive people.

…all of that information certainly helps people that have been newly-diagnosed understand their condition and understand how it's going to affect them and how it's going to affect the others around them. Certainly, it removes much of the stigma because the information contained in that basically tells them that they're not going to die, which previously to reading that information pack, they may have that thought. (Participant 15)

I think it should almost be compulsory that if you're diagnosed with HIV you should have a sit down appointment with a counsellor before you can begin treatment just to make sure that they're okay because people deal with it in really not very good ways and I think the drugs play a big part in coping mechanisms for people who have HIV… (Participant 3)

At a broader level, MSM felt strongly that there is a need for further education and awareness at both the community and medical level to address and breakdown HIV related stigma and to improve support for people living with HIV.

…teach them how to approach people or patients, so those patients don't feel like the stigma, because that's what I felt a little bit with these other people. A bit of pity for you … (Participant 14)

I just hope that over time, the stigma will break down, because I think that's the greatest barrier to ensuring that people are more open about their status and willing to get tested and not living in this fear about how that may affect them … (Participant 7).

## Discussion

In this study MSM’s reactions to their diagnosis varied from shock, devastation and anger to a calm acceptance and feeling HIV would not have a significant impact on their lives. The few MSM who felt they had always engaged in safe sexual behaviors felt particularly perturbed and perplexed by their diagnosis. For a number of MSM the reality of being diagnosed with HIV differed considerably to the idea of having HIV. It is evident that, at a very difficult time emotionally, some MSM feel they have to contend with dealing with their diagnosis in the context of personal distress but also in relation to anticipation of HIV stigma.[24, 25] HIV stigma refers to feeling devalued as a result of internalised negative beliefs (known as internalised stigma), or awareness of societal perceptions about HIV (anticipated stigma) or having experienced discrimination and prejudice because of HIV serostatus (enacted stigma).[26]

MSM who reported strong social support networks, or knew others with HIV, seemed better able to cope with, accept and disclose their diagnosis to others than those with fewer support networks. Social support may take the form of emotional, material, or health-related support.[11] Previous studies have also found that people with HIV who perceive support from particular family and/or friends, as well as anticipate positive outcomes through disclosure of HIV serostatus, are more likely to disclose their status, which can alleviate psychological distress.[13, 27] Of the handful of men who seemed to accept their diagnosis quite well, a few reported little bearing on their day-to-day lives. However, others reported going on sexual ‘rampages’, drug and alcohol binges, breaking up with their partners or making significant changes to their sexual or lifestyle behaviours as a result of their diagnosis. A couple of men also reported feeling depressed in the weeks and months following their diagnosis. Hult and colleagues, in an in-depth study of patients reactions to hearing a positive HIV result, also reported responses varying greatly from being too shocked to comprehend what they were being told to immediately accepting the news and feeling ready for action [28].Overall, most men in our study, regardless of how they felt about their diagnosis, did not want to attribute blame for the transmission of HIV, feeling sexual partners would not have knowingly have transmitted HIV.

Most MSM in our study were selective when disclosing their serostatus, often telling partners perceived to be at risk but no, or only few, close friends. They selectively disclosed their status because of the HIV stigma they felt was still prevalent amongst some family members, the wider community, and at times the medical community, who still harbored ideologies about HIV treatment and prognosis based on outdated information. HIV stigma has been shaped by socio-cultural perceptions, which refers to how the general population may categorise people with HIV, with these perceptions based upon ill-informed beliefs[12] derived from various information sources. The socio-cultural context in which people’s perceptions about HIV/AIDS in Australia have been shaped are linked to a television campaign that aired in 1987. This campaign depicted a ‘Grim Reaper’ character in a 10-pin bowling alley with men, women and children as pins to be struck down by a bowling ball representing HIV.[29] The intention was to communicate to the general population that HIV/AIDS did not discriminate in relation to age or sex. Although it only aired for three weeks, the campaign resulted in widespread hysteria, fear and gross misperceptions about the likelihood of acquiring HIV/AIDS.[30] Recollection of this campaign persists amongst large numbers of the Australian population,[31] and certainly among our participants and their wider family and friends. Even though most MSM in this study actually received social support post-disclosure, the anticipation of experiencing stigma in the form of personal rejection or causing their family distress strongly influenced decisions to disclose. These findings are concerning as they reflect those of studies from a decade ago,[11, 12] suggesting that stigma continues to be associated with fewer disclosures, which may result in less social support, poorer mental health and riskier sex among men newly diagnosed with HIV.[2]

Efforts to address HIV stigma need to continue as a means for improving the social and structural contexts in which MSM receive an HIV diagnosis, disclose their serostatus, and subsequently manage their health.[26] The current Undetectable = Untransmissible (U = U) campaign, based on a range of scientific evidence and which promotes the notion of treatment as prevention, is an excellent example of an approach that is already positively influencing public opinion about HIV [32]. Approaches like this help to remind MSM, their friends and families, that antiretroviral treatment has an excellent record in reducing HIV transmission and decreasing HIV acquisition risk [33]. Prioritising the reduction of stigma may facilitate the disclosure and acceptance of HIV as a manageable condition in the broader community, which would have positive ramifications for HIV prevention and education efforts.[34]

### Strengths and limitations

The main imitation of this study is that most men were recruited from specialist HIV services where they were provided with specialist support. It is likely that the views and experiences of men diagnosed with HIV and managed in other community settings may have differed. It should also be noted that the depth and complexity of data presented in this paper is somewhat limited given the primary purpose of the study was to explore MSM’s partner notification practices and not specifically the factors affecting their acceptance and disclosure of HIV. Finally the difficulties we experienced in recruiting participants and MSM’s concerns around confidentiality also limited the demographic data we were able to collect. Despite these limitations, this study provides an important insight into the factors associated with HIV disclosure and acceptance among newly diagnosed MSM in Australia.

## Conclusion

The prevailing stigma around HIV can have a significant impact on MSM’s acceptance of, and willingness to disclose their HIV serostatus to others, in turn impacting on the levels of professional and social support they receive and the degree to which they accept and cope with their diagnosis.

HIV-related stigma needs to be addressed through community campaigns which better educate both the medical and broader community about the current state of HIV treatment, dispelling old ideologies which only serve to further perpetuate fear and discrimination toward people living with HIV. It is also imperative that persons newly diagnosed with HIV are provided with adequate information, counselling and support both at the time of diagnosis and in the months following diagnosis and provided with safe avenues to meet other people with HIV.
